# Optimizing abrasive wear in sustainable MCC reinforced hemp bamboo epoxy composites for tribological applications

**DOI:** 10.1038/s41598-026-43505-9

**Published:** 2026-03-10

**Authors:** H. D. Supreetha Gowda, Hemaraju, V. G. Pradeep Kumar, B. Suresha, C. R. Rachana, Subraya Krishna Bhat

**Affiliations:** 1 DoS in Computer Science, PG Wing of SBRR Mahajana First Grade College (Autonomous), Pooja Bhagavat Memorial Mahajana Education Centre, Mysuru, 570016 Karnataka India; 2 Department of Mechanical Engineering, BGS Institute of Technology, Adichunchanagiri University, BG Nagara, Nagamangala, Karnataka India; 3 Department of Mechanical Engineering, JSS Science and Technology University, Mysuru, Karnataka India; 4 Manipal Institute of Technology, Manipal Academy of Higher Education, Manipal, India

**Keywords:** Abrasive wear, Box-Behnken Design, Hemp/bamboo hybrid composite, Micro-crystalline Cellulose, Coefficient of friction, Sustainable composite materials, Bio‑based polymer composites, Engineering, Materials science

## Abstract

This study investigates the two-body abrasive wear characteristics of hybrid hemp and bamboo fibers in woven form epoxy (H/B F-Ep) composites reinforced with micro-crystalline cellulose (MCC) using a response surface methodology (RSM) framework and microstructural analysis. The effects of MCC content, emery paper grit, load, and abrading distance, on weight loss, coefficient of friction (CoF), and surface roughness (Ra) were assessed using four factors and three levels using Box–Behnken design. Analysis of variance (ANOVA) was used to develop and statistically validate quadratic regression models, which demonstrated strong predictive ability, a non-significant lack-of-fit, and high coefficients of determination (R² = 95.84–97.06%). Emery paper grit and abrading distance dominate wear loss, MCC content controls frictional response, and both MCC and grit have a substantial impact on surface roughness, according to an ANOVA. Strong nonlinear wear behavior under severe abrasion is indicated by significant interaction and quadratic terms, especially grit^2^ and filler–grit coupling. Optimized MCC loading reduces micro-cutting and stabilizes tribo-layer development, as indicated by main-effects and interaction plots. The statistical results were supported by SEM measurements, which showed a shift from severe micro-ploughing and fiber pull-out in unfilled composites to moderate abrasion and compacted tribo-films at the optimal MCC content. To minimize wear loss (0.0385 g), CoF (0.27), and Ra (1.62 μm), with an overall desirability of 0.96, multi-response desirability optimization determined that 3 wt% MCC, 400-grit abrasive, 150 m abrading distance, and 10 N load were the optimal settings. A strong framework for customizing natural fiber hybrid composites for tribological applications is provided by the combined RSM–SEM technique.

## Introduction

Synthetic fibers such as nylon and polyester are used by manufacturers because of their high performance. However, due to their high energy consumption, inability to decompose, and utilization of micro-polymers, these fibers pose significant environmental challenges^1,2^. Scientists are turning to natural fibers such as flax, jute, sisal, and bamboo to address this problem. These fibers are suitable for structural applications in automobile parts and other items due to their low density, high specific strength, and complete biodegradability. Because of their enhanced stiffness, resistance to wear and corrosion, and higher strength-to-weight ratios, natural fiber-reinforced polymer composites (NF-RPCs), especially those with epoxy matrices, perform exceptionally well in engineering applications^3–7^.

Despite these advantages, NF-RPCs face limitations in tribological performance, specifically in resistance to abrasive wear, which prevents broader industrial adoption. Research on epoxy with ceramic fillers and epoxy-carbon fiber with nano-Al_2_O_3_ fillers, as well as on synthetic fiber-reinforced composites, shows a 13.5% increase in hardness at 2 wt% filler and a decrease in wear loss due to mechanisms such as fiber rupture and micro-ploughing^8,9^. On the other hand, graphite-filled carbon/epoxy composites were used to design lubricating transfer coatings that lower wear rates^10^. In a similar vein, HDPE treated with 5 wt% polybenzimidazole becomes more stable at higher temperatures, enhancing its wear resistance^11^. Natural fiber-reinforced composite systems become more rigid and sticky due to hybridization (e.g., hemp-bamboo, abaca-basalt) and bio-fillers^12^. Bio-fillers, such as pine bark dust, perform better than SiC in three-body wear tests, reaching a specific wear rate of 0.000881 mm^3^/Nm. Important variables include normal load (56.21% contribution), sliding distance, and grit size (up to 76.63% influence). Lubrication also lowers mass loss in jute/rubber composites by 15–44%^13^. Wear mechanisms, including fiber debonding and tribo-film formation, are accurately described by high R^2^ models (92.9% for bagasse-epoxy composites, for example). These models were optimized using bio-inspired algorithms such as SSO (Salp Swarm Optimization), ANOVA for statistical analysis, GRA (Grey Relational Analysis) for multi-objective optimization, and Taguchi techniques for experimental design^14^.

Natural fibers are rarely utilized in many industries due to their poor tribology performance, particularly in terms of resistance to abrasive wear^15^. The technique of combining two or more natural fibers, such hemp and bamboo, to achieve the best possible balance between abrasion resistance and mechanical qualities is known as hybridization^16^. Additionally, a third phase of micro/nano-fillers are being increasingly used to improve important material properties like surface adhesion, wear resistance, and stiffness^17^.

The abrasive wear behavior of NF-RPMCs has been the subject of numerous recent investigations. These studies systematically examine the effects of input parameters like applied load, sliding velocity, abrading distance, and counterface modification on the output parameters of wear rate and friction for a range of fiber types, including abaca^18^, jute^19^, coir^20,21^, hemp^22^, and bamboo^23^. Micron-sized fillers have gained importance and are generally preferable for enhancing the two-body abrasive wear resistance of NF-RPMCs. The primary goals of these fillers are to improve the overall mechanical integrity of the composite and alter the wear mechanisms at the surface. An experimental analysis of jute/natural rubber composites by Mahesh et al.^24^ found that multi-layered green composites perform significantly better than their dry counterparts under abrasive circumstances, with lubricated conditions reducing mass loss by up to 44.44%. In a recent study, Prabhu et al.^25^ investigated the effects of incorporating micron-sized TiO_2_ particles (0–8 wt%) into epoxy composites reinforced with bamboo fibers. According to the study, which was verified using the Taguchi and ANOVA approaches, this filler significantly increases abrasive wear resistance by reducing the specific wear rate. Another study used the Taguchi technique to model how the addition of TiO_2_ micro-filler affected the specific wear rate of epoxy composites reinforced with flax fiber. According to the study, increasing the TiO_2_ filler content significantly enhances the composite’s abrasive wear properties^26^. For epoxy-based composites reinforced with jute, Polyalthia Longifolia, and Mangifera Indica, Heckadka et al.‘s study^27^ demonstrates that normal load is the main driver of two-body wear, accounting for 56.21% of the specific wear rate. Even when sliding speed has negligible effect on deterioration, research using Taguchi’s L16 orthogonal array shows that the transition from fiber-matrix debonding to crater formation remains the primary morphological driver of material loss.

Krishnudu et al.^28^ observed that the fiber loading affects the wear rates of epoxy reinforced with Abutilon indicum fibers. Using Box-Behnken designs, ANFIS models, and SEM analysis, they identified damage such as matrix debonding and microcracking and calculated peak wear at 10 wt% under high loads. According to Darshan et al.^29^, hybrid silk-basalt epoxy nanocomposites’ resistance to three-body abrasion is increased when Halloysite Nanotubes are incorporated. Their Taguchi L27 analysis and ANOVA showed that load and abrading distance are the most important parameters, with the optimal filler levels and sand sizes decreasing wear, as seen by SEM images of surviving fiber structures.

Research on bio-fillers like MCC with hybrid natural fibers (hemp/bamboo) in epoxy matrices is still lacking, especially when it comes to statistically robust designs, even though synthetic composites (such as carbon/glass with micro/nano-fillers or graphite) and certain natural fiber systems (such as abaca-basalt, lubricated jute/rubber) exhibit tribological advancements. Many studies investigate abrasive wear in NF-RPCs, but traditional one-factor-at-a-time approaches ignore interactions, and Response Surface Methodology (RSM) via Box-Behnken Design (BBD), which has been shown to be successful for ANOVA and quadratic modeling, has not been applied extensively to bio-filler hybrid NF-RPCs^30,31^.

Thus, to fill these gaps, this study examines how wear rate and friction in two-body abrasive wear (2-BAW) are affected by fiber types (abaca, jute, coir, hemp, and bamboo), applied load, sliding velocity, abrading distance, and counterface. With the help of RSM-BBD, it presents a unique hemp/bamboo-MCC hybrid in epoxy that allows for predictive tribological improvements for completely sustainable composites.

This work develops a hemp/bamboo hybrid fabric reinforced with MCC filler in an epoxy matrix (H/B F-Ep). Different MCC content, emery grit size, abrading distance, and applied stress are evaluated using two-body abrasive wear (2-BAW). RSM-BBD modeling with ANOVA investigates interaction effects to optimize performance and minimize wear loss in high-performance NF-RPCs. Secondary objectives include model validation for forecasting accuracy and SEM-based wear mechanism analysis. Finally, with improved wear resistance for longer durability, lower maintenance costs (addressing 60% of industrial wear costs), and environmental compliance in structural applications, these optimized composites are aimed at the automotive, aerospace, and construction industries.

## Materials and methods

### Materials

Epoxy resin (LY556, DGEBA equivalent) and hardener (HY951, triethylenetetramine equivalent) were utilized in the matrix at the suggested mixing ratio (e.g., 10:1 by weight).

Natural hemp and bamboo fiber were purchased from Pahartah Fashion LLP, Dharamshala, Himachal Pradesh, India. To improve fiber–matrix interfacial adhesion, hemicellulose, lignin, and surface contaminants were removed from the fibers by treating them with a mild alkali solution (5% NaOH) for 2 h. The secondary reinforcing filler was a bio-filler in the form of microcrystalline cellulose (MCC) powder (20–40 μm particle size, MERCK, Bangalore).

### Composite fabrication

Compression molding was used after the traditional hand lay-up method to produce hybrid composites. The overall fiber content was kept constant at 40 wt% while the hemp and bamboo hybrid layers were placed such that the hemp yarns are oriented in 0^0^ orientations. After the MCC bio-filler was evenly distributed throughout the epoxy matrix by mechanical stirring, it was sonicated for about 30 min at 40 kHz. The hardener was added and vigorously mixed once the dispersion was homogeneous. After that, the mixture was placed into a ready-made mold, and compression molding was done for 24 h at room temperature with an applied pressure of roughly 5 MPa. After that, the matrix was post-cured for three hours at 80 °C to ensure total cross-linking. According to the Box-Behnken Design (BBD), composite samples were made with the designated MCC filler contents (0, 3, and 6 wt%).

### Two-body abrasive wear testing

A Pin-on-Disk tribometer (Magnum Engineers, Bangalore) operated in a modified two-body abrasive configuration (2-BAW) in accordance with ASTM G99 requirements was used to conduct wear testing^32^. With a maximum load and frictional force capacity of 200 N, the device used an EN 31 steel disc (165 mm diameter, 60 HRC) and supported 3–12 mm pins spanning worn track diameters of 10–140 mm. The testing parameters were kept within the 100–2000 RPM speed range, which corresponds to 0.26–12 m/s sliding velocities. To provide consistent contact conditions during testing, composite specimens were machined into pin-shaped samples with nominal dimensions of 25 × 8 × 3.5 mm³. To ensure consistent adherence and remove surface defects, a fresh sheet of silicon carbide (SiC) Emery paper was securely mounted onto the revolving disc prior to each test.

Each specimen’s wear loss was calculated using a high-precision digital analytical balance with a resolution of 0.1 mg by calculating the difference between the initial and final masses. To ensure precision, specimens were ultrasonically cleaned in ethanol and dried before to every weigh-in to eradicate any impurities or adhering material. Following typical error propagation techniques, the standard deviation obtained from triplicate trials was combined with the instrument’s limit of resolution to determine the overall uncertainty in mass loss.

A calibrated force transducer was used to track the friction coefficient (CoF) in real time. The provided values are the steady-state average computed for the last 80% of the abrading distance to remove transitory noise. Estimated at ± 1%, the measurement error takes into consideration the combined impact of mechanical vibrations presents in the tribometer assembly during abrasive contact, sensor resolution, and thermal drift in the load cell.

A portable surface roughness tester (Model: QualiSurf™ II-S, Qualitest USA, Lauderdale) was used to assess the topographical changes brought on by two-body abrasion and characterize the post-test arithmetic mean roughness of the worn surfaces. Measurements were taken at three equally spaced points perpendicular to the sliding direction to take into consideration the directional character of the abrasive grooves and the inherent surface anisotropy. An uncertainty margin, measured by the standard error of the mean across the replicated measurements, is included in the reported Ra values to account for the vertical resolution of the instrument and the spatial variance of the surface texture.

### Design of experiment and response surface methodology

The Box–Behnken design (BBD) was chosen because it generates second-order response surface models more efficiently than full factorial designs (FFD). In contrast to FFD, which necessitates 3^k^ runs (e.g., 81 for four factors at three levels), BBD accomplishes the same goals with just 25–27 trials, thus cutting down on time, reagent use, and analytical expenses. It is specifically designed to capture linear, interaction, and curvature effects in non-linear relationships when fitting quadratic polynomials. Furthermore, unlike 3^k^ FFD or central composite designs (CCD), BBD avoids extreme corner points where all factors are at the same time at their maximum or lowest values, avoiding equipment damage, unstable reactions, or safety issues. For reliable statistical results, its rotatable characteristics to ensure consistent prediction variance throughout the experimental domain.

To examine the input parameters affected the wear loss (Response), a four factor, three level Box-Behnken Design (BBD) was utilized. The different levels and factors utilized to evaluate the abrasive wear behavior are listed in Table 1. Table 2 summarizes the four independent variables together with their actual and coded levels and the output wear loss.

**Table 1:** Factors and levels used to assess the 2-BAW.

Input variables (Units)	Notation	Levels		
		− 1	0	+ 1
Filler (wt%)	A	0	3	6
Emery paper grit (number)	B	220	320	400
AD (m)	C	50	100	150
Load (N)	D	5	10	15

**Table 2:** Experimental design L_27_ results (Box-Behnken Design).

SL. No	Filler (A)	Load (D)	Abrading distance (c)	Emery paper (B)	Wear loss	CoF	Ra
1	0	10	150	320	0.0908	0.47	2.61
2	3	15	50	320	0.054	0.41	2.22
3	0	5	50	320	0.0501	0.51	2.92
4	3	5	100	400	0.0307	0.42	2.22
5	6	10	50	320	0.0565	0.33	1.72
6	6	15	100	320	0.0834	0.32	1.65
7	3	10	100	320	0.0612	0.43	2.25
8	0	15	100	320	0.0492	0.45	2.52
9	3	10	150	220	0.2108	0.43	2.25
10	3	10	100	320	0.0698	0.44	2.26
11	6	10	100	400	0.0376	0.27	1.52
12	3	10	50	220	0.1045	0.44	2.61
13	3	10	50	400	0.0321	0.40	2.21
14	6	10	100	220	0.2412	0.35	2.02
15	6	10	150	320	0.1119	0.32	1.51
16	3	5	150	320	0.0801	0.39	2.31
17	3	15	100	400	0.0418	0.37	2.28
18	3	5	100	220	0.164	0.45	2.61
19	0	5	100	320	0.0689	0.51	2.82
20	0	10	100	400	0.0403	0.44	2.31
21	3	10	150	400	0.048	0.38	2.28
22	3	10	100	320	0.0799	0.40	2.21
23	3	5	50	320	0.0424	0.42	2.31
24	6	5	100	320	0.0639	0.34	1.83
25	3	15	150	320	0.0725	0.35	2.24
26	0	10	100	220	0.1608	0.49	2.93
27	3	15	100	220	0.1923	0.43	2.41

## Results and discussion

### Response surface methodology

To assess the interactions between the chosen process parameters and establish a predicted correlation between the independent variables and the measured responses, Response Surface Methodology (RSM) was utilized. Analysis of variance (ANOVA) was used to statistically validate the created regression models to determine their significance and relevance.

Wear loss is the response variable in the 27 tests in the Box-Behnken design indicated by Table 3. Hypotheses are tested using ANOVA, and P-values less than 0.05 at the 95% confidence level indicate statistical significance. With R^2^ = 97.06%, R^2^ (adjusted) = 93.62%, and R^2^ (predicted) = 82.32%, the model exhibits a strong match.

**Table 3:** Analysis of variance table for wear loss.

Source	DF	Adj SS	Adj MS	F-Value	*P*-Value	% Contribution
Model	14	0.083289	0.005949	28.25	0.000	
Linear	4	0.067937	0.016984	80.65	0.000	
Filler	1	0.001836	0.001836	8.72	0.012	2.14
Applied load	1	0.000150	0.000150	0.71	0.415	0.17
Abrading distance	1	0.006795	0.006795	32.27	0.000	7.92
Emery paper	1	0.059235	0.059235	281.27	0.000	69.03
Square	4	0.007667	0.001917	9.10	0.001	
Filler * Filler	1	0.000384	0.000384	1.82	0.202	0.45
Applied load *Applied load	1	0.000127	0.000127	0.60	0.452	0.15
Abrading distance * Abrading distance	1	0.000099	0.000099	0.47	0.507	0.12
Emery paper * Emery paper	1	0.005235	0.005235	24.86	0.000	6.10
2-Way Interaction	6	0.004855	0.000809	3.84	0.023	
Filler * Applied load	1	0.000543	0.000543	2.58	0.134	0.63
Filler * Abrading distance	1	0.000048	0.000048	0.23	0.642	0.06
Filler * Emery paper	1	0.001866	0.001866	8.86	0.012	2.17
Applied load * Abrading distance	1	0.000052	0.000052	0.25	0.629	0.06
Applied load * Emery paper	1	0.000101	0.000101	0.48	0.502	0.12
Abrading distance * Emery paper	1	0.002182	0.002182	10.36	0.007	2.54
Error	12	0.002527	0.000211			2.95
Lack-of-Fit	10	0.002352	0.000235	2.68	0.302	
Pure Error	2	0.000175	0.000088			
Total	26	0.085816				

**S** 0.0145121 **R-sq** 97.06% **R-sq(adj)** 93.62% **R-sq(pred)** 82.32%.

Emery paper grit is the primary cause of wear loss, accounting for 69.03% (F = 281.27, *P* = 0.000), significantly more than any other factor. At 7.92% (F = 32.27, *P* = 0.000), abrading distance comes in second, followed by the emery paper^2^ quadratic term at 6.0% (F = 24.86, *P* = 0.000). Filler * emery paper (2.17%, F = 8.86, *P* = 0.012) and abrading distance * emery paper (2.54%, F = 10.36, *P* = 0.007) are significant interactions. 2.14% comes from filler alone (F = 8.72, *P* = 0.012). Most terms (*P* > 0.05) and applied load (0.17%, *P* = 0.415) are considered nonsignificant^33^. Together, linear terms account for 79.26% of the total, with squares accounting for 8.92% and interactions for 5.66%. Corresponding to abrasive wear behavior in polymer and hybrid composites under two-body abrasion, wear loss increases with applied load and coarser emery paper (lower grit). Emery paper grit is the primary cause of wear, which is consistent with research showing that grit size much exceeds load or speed in polymer composites. Its harshness encourages micro-cutting on abrasive tracks. Similar dominance is seen in copper-alumina composites, where wear severity increases with abrading distance/load, with grit as the main factor by ANOVA^34^. While load has no discernible effect (*P* = 0.415), emery paper grit has the most impact (69.03% contribution), followed by abrading distance (7.92%). In fiber-reinforced polymers, higher loads increase contact pressure and friction, which encourages micro-cutting, ploughing, and debris formation; however, grit size and distance are more important in this situation, as demonstrated by polyethylene and nanofiller composites^35^.

With frictional heating and surface softening getting worse at greater distances, particularly on coarse emery paper, abrading distance directly scales wear loss; their interaction (2.54% contribution, *P* = 0.007) validates synergy^36^. This is consistent with Box-Behnken investigations in emery paper abutilon-epoxy composites, where load and distance variations revealed increased specific wear rates of up to 2.9 × 10^− 12^ mm³/N-m^28^.

Additionally, load and grit were identified as the main contributors (~ 69%) in hybrid nanofiller epoxy composites, with ANOVA indicating quadratic and interaction significance like Table 3. Like research on Al–SiC and natural fiber–epoxy, the emery paper² term (6.0% contribution, *P* = 0.000) shows nonlinearity, with coarse grits amplifying wear disproportionately^37^. At ideal loads, fillers strengthen resistance to strong abrasives, according to the filler * emery paper interaction (2.17% contribution, *P* = 0.012). Debris clogging may stabilize rates later, as in Kevlar- and natural-fiber hybrids, even when wear increases with distance; the model’s high R^2^ values support a strong prediction of these trends^38^. Filler-grit synergies were validated by statistical evaluation of nanoclay-filled PA66/PP blends, which showed decreased three-body abrasive wear with distance/load^39^. Strong prediction is indicated by high R^2^ values (97.06%, 93.62% corrected), which are in line with RSM-ANOVA in AlCrN-coated wear experiments that use Box-Behnken for load/speed/distance optimization^40^.

The Box-Behnken design (BBD) with 27 tests is shown in Table 4, with the response variable being the coefficient of friction (CoF). Statistical significance is evaluated using analysis of variance (ANOVA), and at the 95% confidence level, significant effects are indicated by P-values < 0.05. With R^2^ = 95.84%, R^2^ (adjusted) = 90.98%, and R^2^ (predicted) = 80.26%, the model shows excellent fit^41^.

**Table 4:** ANOVA for CoF.

Source	DF	Adj SS	Adj MS	F-Value	*P*-Value	% Contribution
Model	14	0.090329	0.006452	19.74	0.000	
Linear	4	0.080356	0.020089	61.45	0.000	
Filler	1	0.065763	0.065763	201.15	0.000	69.77
Applied load	1	0.003303	0.003303	10.10	0.008	3.50
Abrading distance	1	0.001795	0.001795	5.49	0.037	1.90
Emery paper	1	0.008008	0.008008	24.50	0.000	8.50
Square	4	0.001668	0.000417	1.28	0.333	
Filler * Filler	1	0.001481	0.001481	4.53	0.055	1.57
Applied load *Applied load	1	0.000218	0.000218	0.67	0.430	0.23
Abrading distance * Abrading distance	1	0.000569	0.000569	1.74	0.212	0.60
Emery paper * Emery paper	1	0.000385	0.000385	1.18	0.299	0.41
2-Way Interaction	6	0.001234	0.000206	0.63	0.705	
Filler * Applied load	1	0.000541	0.000541	1.65	0.223	0.57
Filler * Abrading distance	1	0.000044	0.000044	0.14	0.719	0.05
Filler * Emery paper	1	0.000220	0.000220	0.67	0.428	0.23
Applied load * Abrading distance	1	0.000177	0.000177	0.54	0.476	0.19
Applied load * Emery paper	1	0.000220	0.000220	0.67	0.428	0.23
Abrading distance * Emery paper	1	0.000038	0.000038	0.12	0.739	0.04
Error	12	0.003923	0.000327			4.16
Lack-of-Fit	10	0.003056	0.000306	0.71	0.713	
Pure Error	2	0.000867	0.000433			
Total	26	0.094252				

**S** 0.0180812 **R-sq** 95.84% **R-sq(adj)** 90.98% **R-sq(pred)** 80.26%.

At 69.77% contribution (F = 201.15, *P* = 0.000), filler content dominates CoF, outweighing other linear variables such as abrading distance (1.90%, F = 5.49, *P* = 0.037), applied load (3.50%, F = 10.10, *P* = 0.008), and emery paper (8.50%, F = 24.50, *P* = 0.000). While quadratic terms (1.28 overall F-value, *P* = 0.333) and 2-way interactions (*P* = 0.705) are not significant (*P* > 0.05), all linear terms are significant (overall *P* = 0.000), indicating primarily linear behavior. The contribution of error is negligible (4.16%), and the lack-of-fit is not statistically significant (*P* = 0.713)^42,43^.

The highly significant regression model for CoF (model F = 19.74, *P* = 0.000) confirms that frictional variability is driven by filler, load, distance, and emery paper. The primary function of filler is to increase surface hardness and interface stability while decreasing asperity deformation. While load and distance increase real contact area and raise interface temperatures, the texture of emery paper modifies contact interactions^44^.

The linear dominance within the investigated ranges is reinforced by quadratic effects such as filler^2^, which approach borderline significance (*P* = 0.055, 1.57% contribution) but are still negligible overall. This is consistent with tribological principles, which state that sliding friction in composites is determined by surface roughness and material composition^45,46^. The model’s predictive power for maximizing frictional performance is demonstrated by its low error and strong R^2^ values.

Filler has the greatest impact on the coefficient of friction (CoF) (69.77%). While emery paper (8.50%) and applied load (3.50%) have moderate effects, abrading distance only accounts for 1.90% of the effects. Overall, the model covers 95.84% of the total variation with a continuously low error (4.16%), while quadratic terms and interactions are insignificant (< 3% total).

The Box-Behnken design with 27 tests and surface roughness (Ra) as the response variable is shown in Table 5. With R^2^ = 96.04%, R^2^(adjusted) = 91.41%, and R^2^(predicted) = 77.45%, the model exhibits high fit, and ANOVA assesses significance at *P* < 0.05 (95% confidence)^47^.

**Table 5:** ANOVA for surface roughness (Ra).

Source	DF	Adj SS	Adj MS	F-Value	*P*-Value	% Contribution
Model	14	3.54282	0.25306	20.76	0.000	
Linear	4	2.99089	0.74772	61.34	0.000	
Filler	1	2.50241	2.50241	205.29	0.000	67.84
Applied load	1	0.07185	0.07185	5.89	0.032	1.95
Abrading distance	1	0.03469	0.03469	2.85	0.117	0.94
Emery paper	1	0.33667	0.33667	27.62	0.000	9.13
Square	4	0.14069	0.03517	2.89	0.069	
Filler * Filler	1	0.05558	0.05558	4.56	0.050	1.51
Applied load *Applied load	1	0.02639	0.02639	2.16	0.167	0.72
Abrading distance * Abrading distance	1	0.00005	0.00005	0.00	0.950	0
Emery paper * Emery paper	1	0.02513	0.02513	2.06	0.177	0.68
2-Way Interaction	6	0.08165	0.01361	1.12	0.408	
Filler * Applied load	1	0.01200	0.01200	0.98	0.341	0.33
Filler * Abrading distance	1	0.00315	0.00315	0.26	0.620	0.09
Filler * Emery paper	1	0.00248	0.00248	0.20	0.660	0.07
Applied load * Abrading distance	1	0.00328	0.00328	0.27	0.613	0.09
Applied load * Emery paper	1	0.01469	0.01469	1.20	0.294	0.40
Abrading distance * Emery paper	1	0.04824	0.04824	3.96	0.070	1.31
Error	12	0.14627	0.01219			3.97
Lack-of-Fit	10	0.14487	0.01449	20.70	0.047	
Pure Error	2	0.00140	0.00070			
Total	26	3.68910				

**S** 0.0110405 **R-sq** 96.04% **R-sq(adj)** 91.41% **R-sq(pred)** 77.45%.

Filler is the most common, contributing 67.84% (F = 205.29, *P* = 0.000), followed by applied load (1.95%, F = 5.89, *P* = 0.032) and emery paper (9.13%, F = 27.62, *P* = 0.000). There is no significance in abrading distance (0.94%, *P* = 0.117). Overall effects (*P* = 0.000) are driven by linear terms, although quadratics (*P* = 0.069), led by filler^2^ (1.51%, *P* = 0.050), approach significance; interactions are still small (*P* = 0.408), while distance*emery approaches relevance (1.31%, *P* = 0.070). Higher-order refinements are suggested by the significant lack-of-fit (*P* = 0.047), despite the low error (3.97%).

The majority of variance in the complete model is explained by linear factors, and it is highly significant (F = 20.76, *P* = 0.000). The main reason filler is preferred is probably because its increased composite hardness lowers Ra during abrasion^48^. Emery paper uses grit texture to control asperity interactions, and load somewhat increases contact area; distance has very little direct effect.

At filler extremes, quadratic curvature appears (*P* = 0.050), however interactions are not very important. The substantial lack-of-fit suggests that the quadratic model overlooks some nonlinearity, which is typical in wear studies where cubic terms are needed. Despite limited predictability, a high R^2^ indicates a strong association. Ra is mostly controlled by filler (67.84%), with emery paper (9.13%) acting as a secondary factor. Distance is negligible, whereas load (1.95%) adds a moderate amount of input. Interactions and quadratics account for less than 5% of the total. The model captures 96.04% of the variation and has a low error (3.97%).

**Fig. 1:**
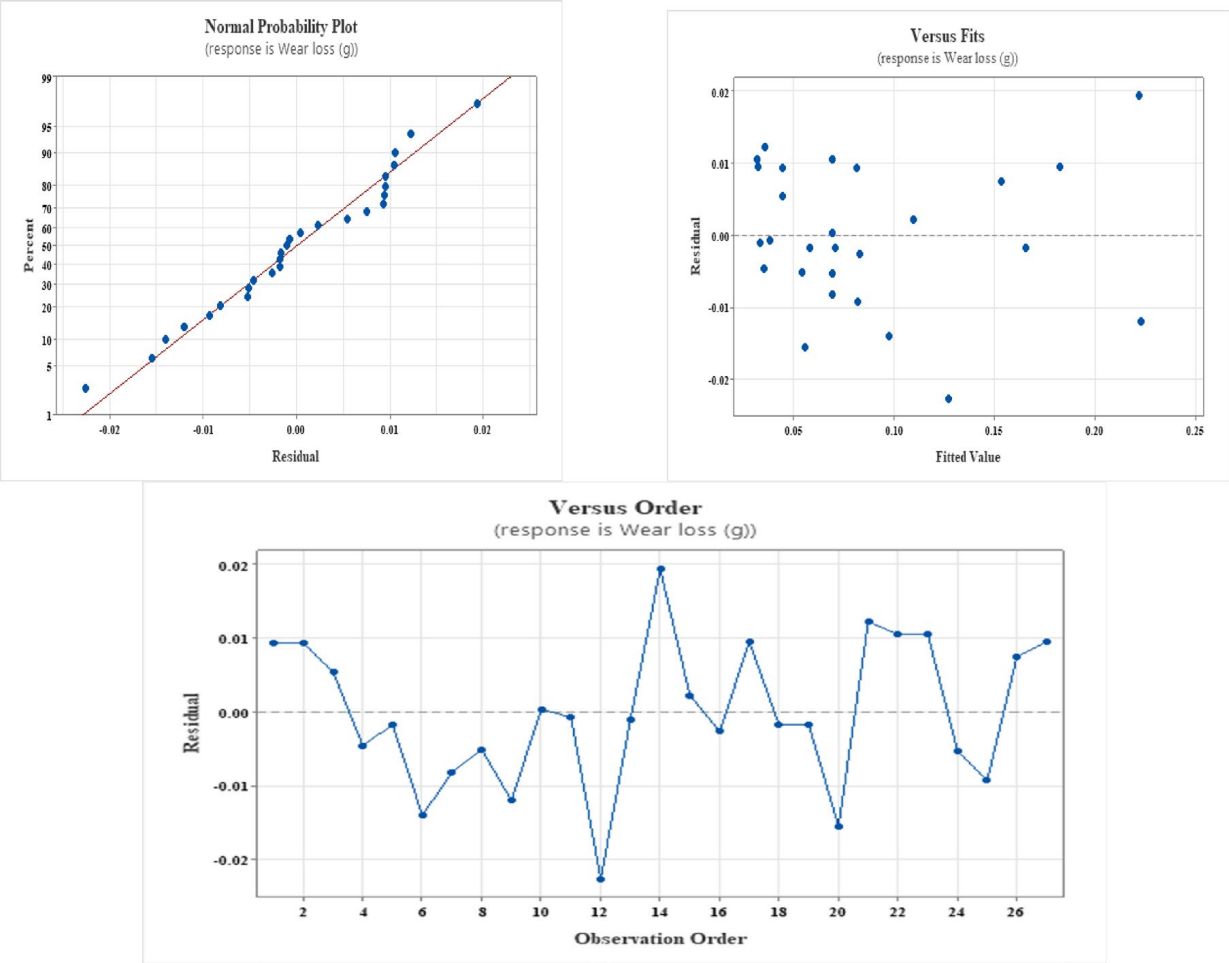
Residual plots for wear loss.

**Fig. 2:**
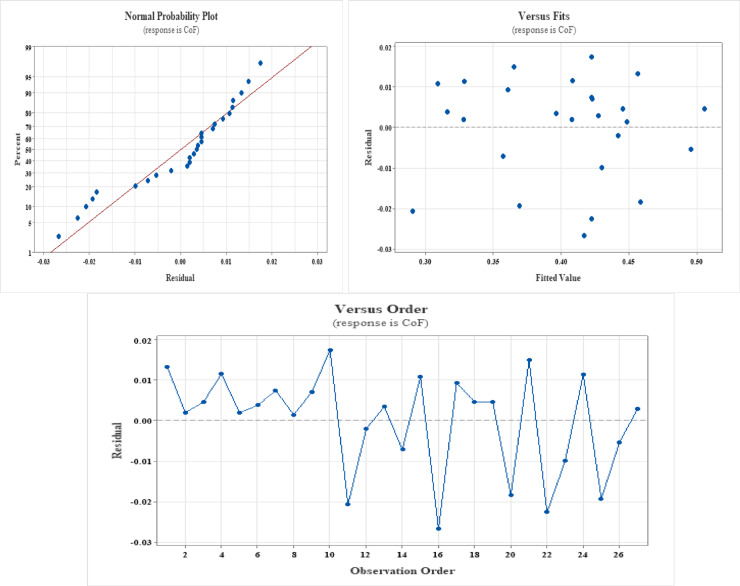
Residual plots for CoF.

**Fig. 3:**
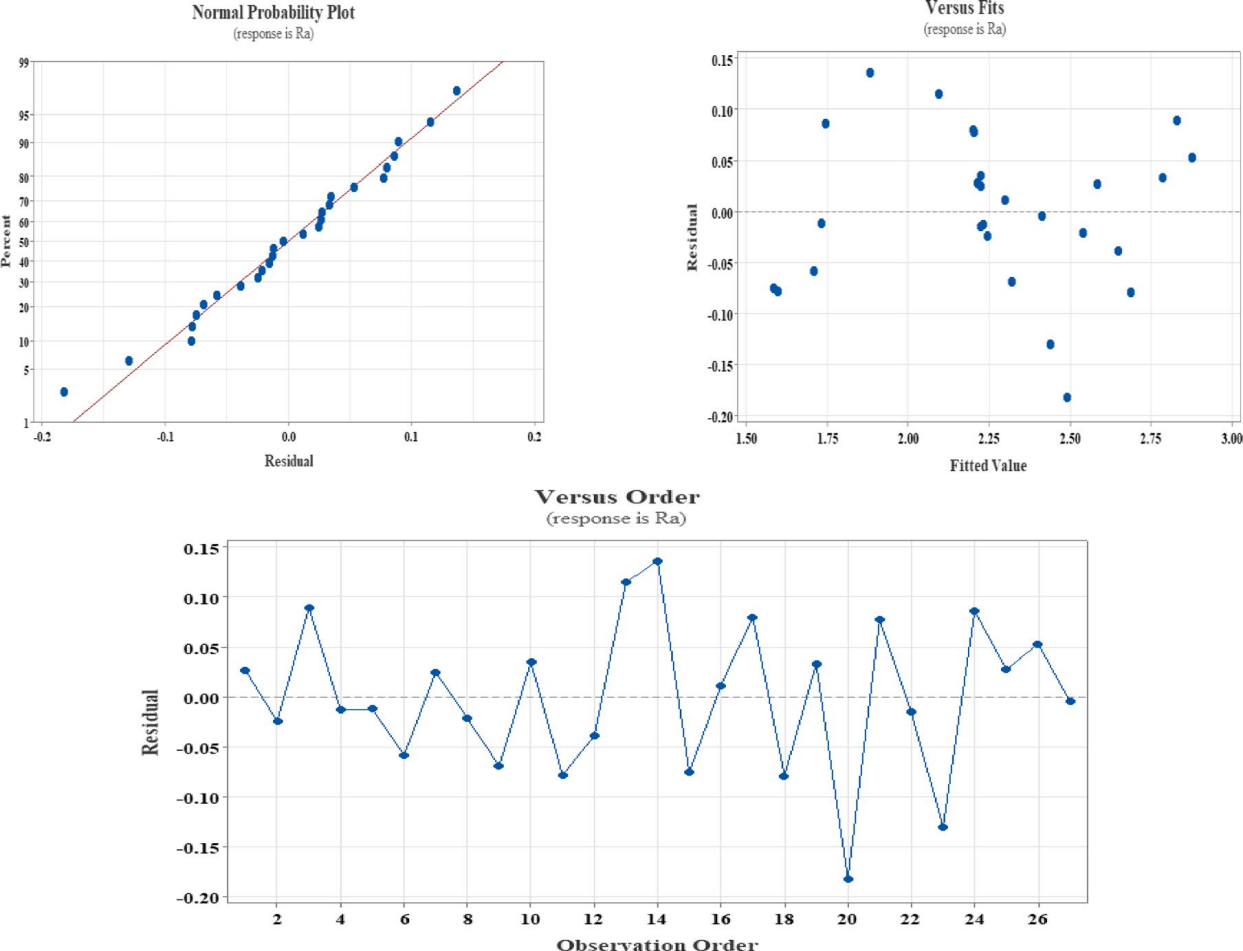
Residual plots for surface roughness (Ra).

The residuals hardly vary from normality and are closely aligned with the fitted axis, as seen in Figs. 1 and 2, and 3. A strong linear link between the influencing factors and the response variable is confirmed by the residuals’ near-normal distribution. The residuals in the remaining plots are spread randomly, which is a prerequisite for a high degree of agreement between the projected values and the experimental data.

### Main effects plot analysis on two-body abrasive wear

**Fig. 4:**
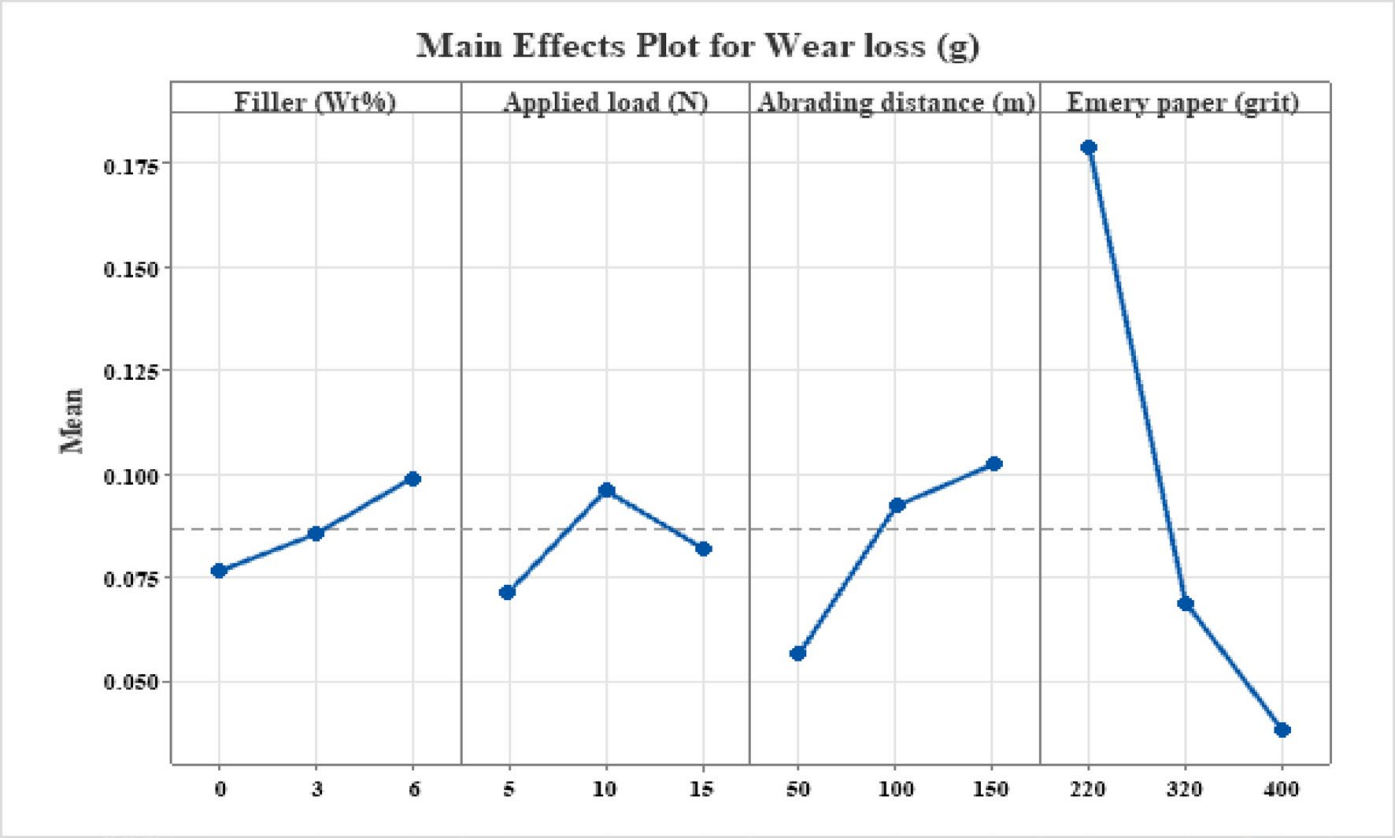
Main effect plot for wear loss.

Figure 4 displays the main effects plot for wear loss of hybrid hemp and bamboo hybrid fiber-epoxy (MCC-H/B F-Ep) composites filled with MCC, highlighting the distinct effects of filler content, abrasive grit size, load, and abrading distance. It is evident from the slope and amount of the mean response that the wear behavior is dominated by abrasive grit size and abrading distance, followed by applied load and filler content.

#### Effect of MCC loading

The positive slope of the major impacts plot indicates a small increase in wear loss as MCC content rises from 0 to 6 wt%. Although MCC particles are known to increase surface hardness and stiffness at appropriate loadings, excessive filler addition can compromise interfacial integrity due to particle agglomeration and inadequate epoxy matrix wetting. Increased wear loss in the existing hybrid system with higher MCC content suggests that MCC agglomerates act as stress concentrators, filler pull-out occurs under abrasive action, and detached MCC particles contribute to third-body abrasion. Similar filler-induced wear escalation above optimal content has been well reported for particulate-filled polymer composites subjected to abrasive wear conditions^49,50^. Recent research^51–54^ has shown that soft fillers, such as shell powders or low-modulus hybrids, can reduce the abrasion resistance of natural fiber-reinforced epoxy composites by promoting poor dispersion, weak interfacial bonding, and increased matrix deformation under load.

#### Effect of applied load

Wear loss first rises with applied load up to 10 N, after which it slightly decreases at 15 N. Higher contact pressure, which encourage deeper abrasive penetration, matrix micro-cutting, and fiber–matrix debonding, are responsible for the first increase. The compaction of wear debris to produce a mechanically stable tribo-layer, greater actual contact area which distributes the stress more evenly, and partial load sharing by the woven hemp-bamboo reinforcement could all contribute to the modest decrease in wear loss at higher loads. Biswas et al.^54^ found that, although having worse mechanical properties, alumina-filled glass fiber-epoxy composites are more resistant to erosion than their red mud-filled counterparts. Micro-ploughing mechanisms were shown by SEM, and maximal erosion occurred at a 60° impingement angle. The dominance of filler content (67.84% for Ra and 69.77% for CoF in Tables 3, 4 and 5) is highlighted in alignment by our analysis, indicating semiductile behavior under abrasive conditions. Emery paper grit highlights the best hard fillers for tribological improvement by paralleling erodent size effects. Also, natural fiber-reinforced epoxy systems have shown this kind of non-monotonic load dependency, where fiber bridging effects and debris compaction become important at higher loads^55^. Recent studies demonstrates that soft fillers weaken the abrasion resistance of natural fiber-reinforced epoxy composites, and that applied load exacerbates this by increasing interfacial shear and matrix deformation. Mia et al. discovered, for example, that soft nano-clay fillers in banana-epoxy increased wear rates under higher loads because they did not transfer load well^56^. Similarly, soft wood-apple shell fillers exacerbated three-body abrasion under high pressures, peaking at 45–60° impingement, according to Sienkiewicz et al.^57^. According to Taguchi analysis, Davis Hans et al. found that the soft filler microcrystalline cellulose in hemp/bamboo epoxy increased specific wear by 25–40% at loads greater than 20 N^58^.

#### Effect of abrading distance

As the abrading distance increases, wear loss clearly increases, indicating cumulative material removal. The wear process is dominated by mild abrasion with minimal matrix degradation at shorter distances (50 m). The MCC-modified epoxy surface deteriorates, hemp and bamboo fibers are exposed and fractured, and the matrix is gradually removed as the sliding distance rises due to recurrent contact with abrasive asperities. For structural tribological applications, the near-linear trend indicates steady-state wear without catastrophic surface breakdown. For lignocellulosic fiber–epoxy composites under two-body abrasion, similar distance-dependent wear behavior has been documented^59,60^.

#### Effect of SiC emery paper grit

The impact on abrasive grit size is the most noticeable. As the grit number increases (from 220 to 400), wear loss drops dramatically, suggesting that coarser abrasives cause noticeably more wear. Sharp asperities result in severe micro-ploughing and cutting of the epoxy matrix, fiber fracture and pull-out, and rapid MCC particle detachment at lower grit numbers (coarser abrasives). Finer grits, on the other hand, encourage surface polishing rather than forceful cutting and create shallow grooves. The wear mechanism shifts from severe abrasion to moderate abrasion as grit size increases, as confirmed by this strong grit dependence^48^. This trend has been well documented in polymer and natural fiber composite systems.

Recent investigations have shown that soft fillers reduce the abrasion resistance of natural fiber-reinforced epoxy composites. In contrast, finer SiC emery paper grit (reduced size) paradoxically shows soft phases by moving from forceful cutting to polishing that reveals subsurface faults. Longer abrading lengths accelerate wear by creating cumulative frictional heating and fatigue failure at weak interfaces. ANFIS modeling confirmed distance-grit synergy degrading low-fiber hybrids^28^. When the hemp/bamboo epoxy slid over fine grits for a long time, Davis Hans et al.^58^ found that the microcrystalline cellulose (soft filler) increased specific wear by 30%. Through machine learning optimization, they ascribed this to debris compaction and thermal softening. Through ASTM G99 testing, Shettahalli et al.‘s^61^ study confirms that filler content dominates ATS-treated flax fabric-epoxy composites (0–45 wt% fiber), where greater fiber loading (45-TFE) reduced wear loss, SWR, and COF under various loads and grits.

### Main effects plot analysis of coefficient of friction

**Fig. 5:**
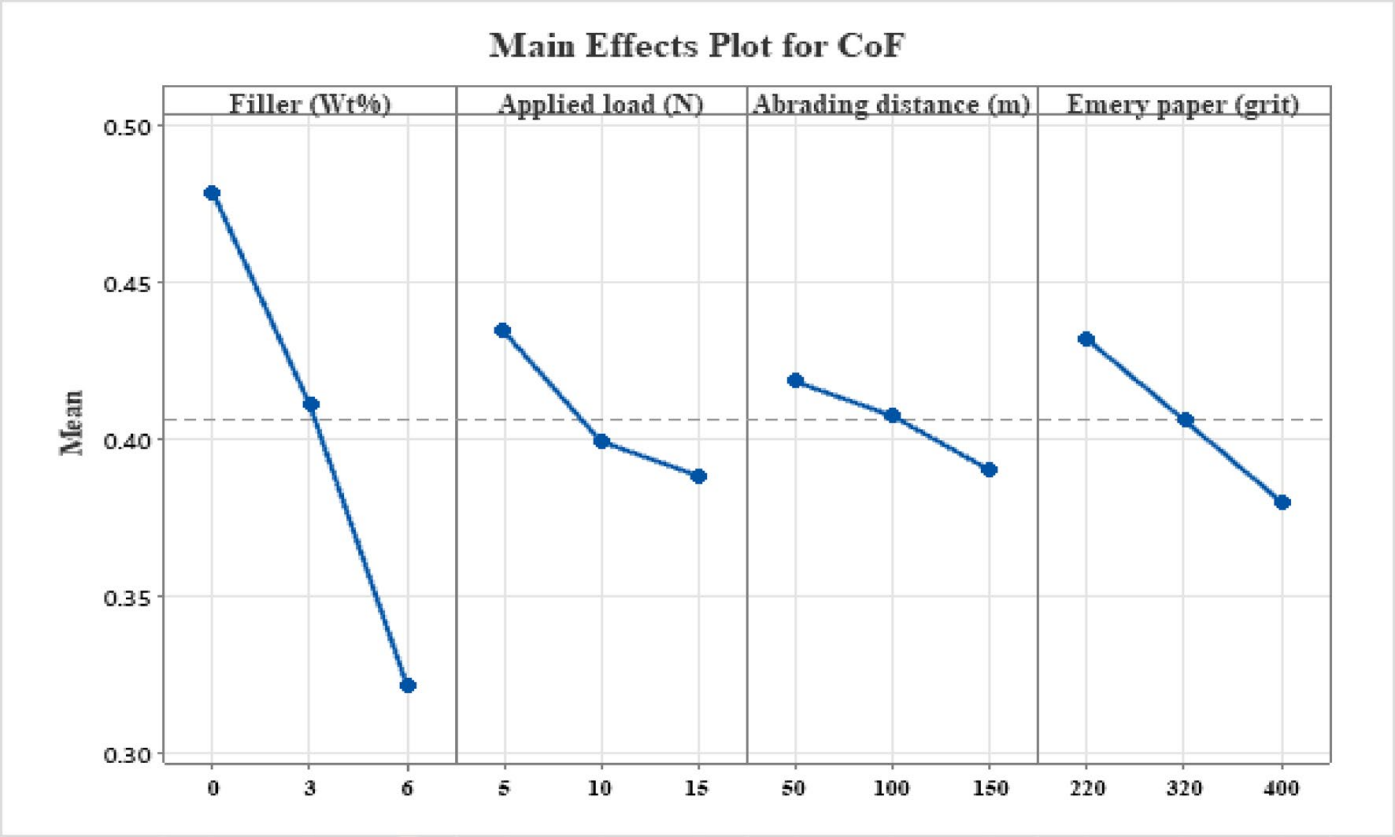
Main effect plot for CoF.

The main effects plot for CoF of MCC-filled H/B F-Ep composites is shown in Fig. 5. The plot clearly shows that MCC filler content has the largest effect on CoF, followed by abrading distance, applied force, and abrasive grit size. The intricate connections between surface chemistry, tribo-layer development, and fiber–matrix interactions are reflected in the general patterns.

#### Effect of MMC content

As the filler loading increases from 0 to 6 wt%, a noticeable drop in CoF is seen. This notable decrease implies that MCC functions within the epoxy matrix as an efficient friction-modifying agent. Finely distributed MCC particles decrease direct asperity contact between the counterface and the composite surface and encourage the development of a stable transfer coating. Furthermore, during sliding, MCC particles may experience modest shear-induced fragmentation, resulting in lubricious debris that reduces interfacial shear strength. Recent tribological studies have demonstrated similar friction-reducing effects of cellulose-based fillers in polymer composites, especially under dry sliding circumstances^62,63^.

#### Effect of applied load

As the applied load increases from 5 to 15 N, the CoF gradually drops. The development of a mechanically stable tribo-layer at greater stress is frequently cited as the cause of this inverse relationship. Increased normal force increases the real contact area and encourages debris compaction, which lowers frictional resistance and functions as a solid lubricant. Additionally, by limiting excessive matrix deformation and promoting load sharing, the hybrid hemp-bamboo woven architecture stabilizes the friction response. Natural fiber-reinforced epoxy composites and bio-filled polymer systems have shown similar load-dependent CoF decrease^64,65^.

#### Effect of abrading distance

The main effects plot shows a progressive drop in CoF with increasing abrading distance. Surface imperfections and loosely bound MCC particles increase friction during the early stages of sliding. Reduced resistance to motion results from surface smoothing and transfer layer stabilization while sliding continues. The enhanced interfacial compatibility between the composite surface and the abrasive counterface is reflected in this trend, which shows a shift from a running-in regime to a steady-state friction regime. For lignocellulosic fiber composites under dry sliding circumstances, comparable distance-dependent friction stabilization has been documented^66^.

#### Effect of emery paper grit

As the grit number increases (from 220 to 400), the CoF significantly drops, indicating that coarser abrasives cause more friction because of vigorous ploughing and micro-cutting. Sharp abrasive asperities improve interlocking and sliding resistance at smaller grit levels, which raises CoF. Finer grits, on the other hand, encourage surface cleaning and lessen asperity penetration depth, which lowers friction. This substantial grit dependence is consistent with current research on abrasive-induced friction processes and is well-documented in polymer composite tribology^67,68^.

The formation of MCC-assisted transfer films, debris compaction and tribo-layer stabilization, decreased asperity interlocking at finer grit sizes, and load-assisted surface conformity improved by hybrid woven reinforcement appear to be the main friction-reduction mechanisms, according to the observed CoF trends. Hemp-bamboo hybrid mats and MCC filler work in concert to stabilize friction during extended sliding.

### Main effects plot analysis on surface roughness

**Fig. 6:**
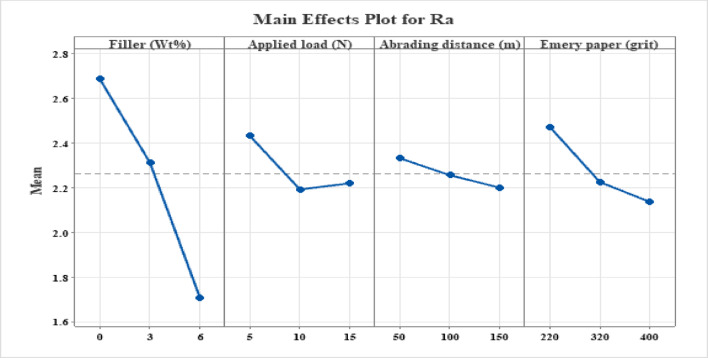
Main effect plot for surface roughness (Ra).

The surface roughness (Ra) main effects plot of MCC-filled hybrid hemp-bamboo woven epoxy composites are displayed in Fig. 6. The plot shows that abrasive grit size and MCC filler content have the most effects on post-wear surface roughness, followed by applied load and abrading distance. The combined impacts of fiber–matrix interaction, abrasive severity, and matrix change by MCC are reflected in the reported trends.

#### Effect of MCC filler content

As the MCC concentration increases from 0 to 6 wt%, there is a noticeable decrease in Ra. Due to significant matrix ploughing and exposed fiber ends during abrasion, the unfilled composite has the maximum surface roughness. Worn surfaces become smoother as the amount of MCC added increases because the epoxy matrix is more resilient to plastic deformation. The production of a compacted tribo-layer containing MCC debris, improved load sharing at the surface, decreased depth of abrasive grooves, and increased matrix stiffness and hardness as a result of MCC dispersion are all responsible for the decrease in roughness. Recent tribological experiments have found similar roughness reduction with bio-based or particle fillers in polymer composites, where optimal filler loading encouraged surface smoothing during abrasion^60,69^.

#### Effect of applied load

When the applied stress increases from 5 to 10 N, surface roughness significantly decreases before slightly increasing at 15 N. Increased contact pressure at moderate loads encourages wear debris compaction and surface conformance, resulting in smoother wear tracks. However, localized fiber breakage, matrix cracking, and micro-delamination start to happen at greater loads, which causes Ra to significantly increase. Although severe surface damage is postponed by the hybrid hemp-bamboo woven architecture, high load nevertheless encourages roughening through interfacial failure and fiber pull-out. Natural fiber and polymer composites under dry sliding have similarly shown this non-linear load dependency of surface roughness, with damage processes predominating at higher loads and debris compaction dominating at intermediate values^70^.

#### Effect of abrading distance

As the abrading distance rises, the composite’s average surface roughness (Ra) gradually decreases, indicating a progressive smoothing of the contact interface. Acute surface asperities in the early stages of abrasion and loosely attached lignocellulosic fibers are the causes of elevated roughness levels. But as sliding proceeds, these unstable characteristics are sheared away by the frequent contact with the abrasive counterface, which promotes the formation of a stable tribo-layer. At this point, the initial high-wear regime gives way to a steady-state phase with a more homogeneous and smooth surface topography. This distance-dependent behavior has been confirmed by recent research on epoxy systems reinforced with lignocellulosic^60,61^. While Kumar et al.^71^ demonstrated that compacted tribofilm formation, enhanced by natural lignin and fillers, smoothens the surface and stabilizes friction with increasing sliding distance, Kuram^72^ reported an initial running-in degradation followed by surface stabilization at wear equilibrium.

#### Effect of emery paper grit

As the grit number increases from 220 to 400, the surface roughness (Ra) significantly decreases because finer grits encourage surface polishing and shallow micro-scratching, while coarser abrasives cause severe micro-cutting, deep grooves, and localized fiber damage. In line with the findings of Zaghloul et al.^73^, who reported a shift from aggressive material removal to surface smoothing with decreasing abrasive particle size, this grit-dependent response validates abrasive size as a major driver of surface evolution in polymer and natural fiber composites.

### Interaction plots

#### Interaction effects on wear loss

**Fig. 7:**
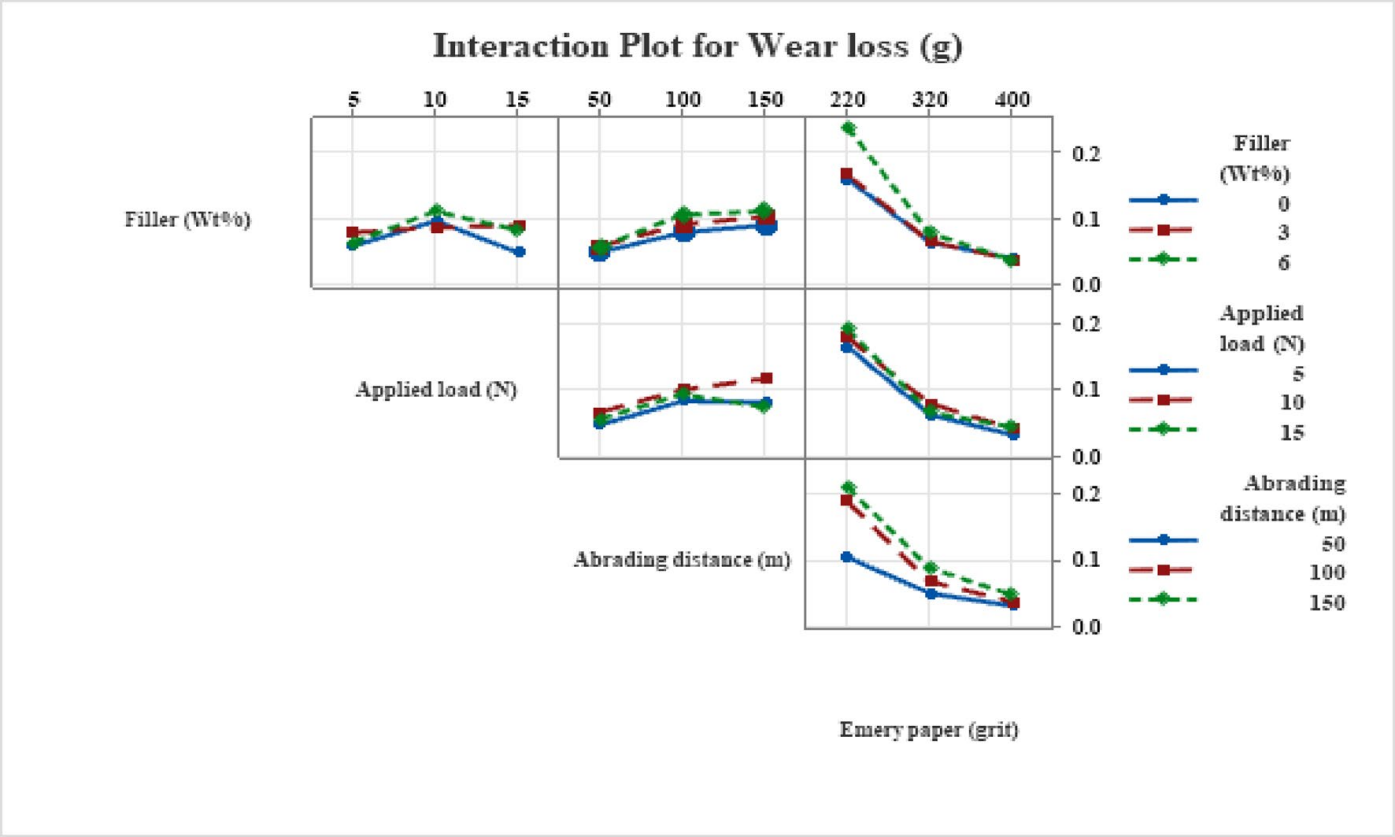
Interaction plot for wear loss.

The interaction effects of MCC filler content with Emery paper grit, load, and abrading distance on the weight loss of hybrid H/B F-Ep composites are shown in Fig. 7. Wear behavior is controlled by linked parameter effects rather than separate influences, according to the non-parallel interaction patterns.

MCC filler content and abrasive grit clearly interact, with unfilled composites showing significantly higher wear at coarse grit (220), while MCC-filled systems show significantly lower wear because of filler-assisted matrix strengthening and debris compaction that prevent severe micro-cutting. The interaction decreases at finer grits (320–400), suggesting that abrasive severity predominates over filler effects.

In contrast to MCC-filled systems, which exhibit a muted response due to enhanced load-bearing capacity and stress redistribution via the hybrid weave design, the filler–load interaction shows that wear loss accelerates quickly with load in unfilled composites. Similarly, the filler-abrading distance interaction demonstrates that the stabilization of a compacted MCC-rich tribo-layer in MCC-filled composites considerably reduces wear progression with sliding distance. The overall effect of MCC filler and hybrid fabric in minimizing wear in abrasive and mechanical circumstances is seen in Fig. 7.

#### Interaction effects on coefficient of friction

**Fig. 8:**
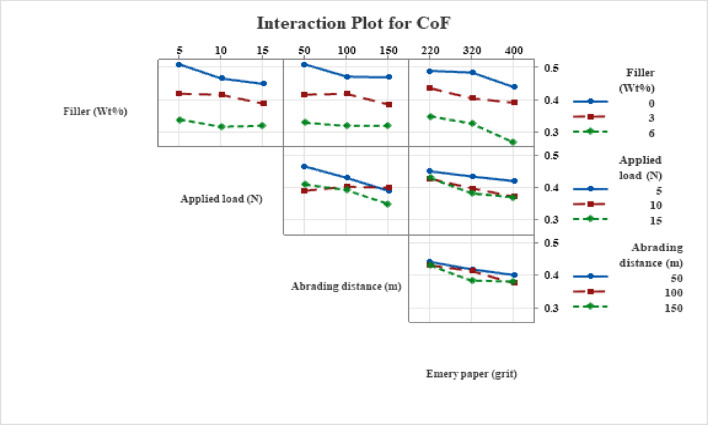
Interaction plot for CoF.

The interaction effects of MCC filler content with applied load, abrasive grit, and abrading distance on the coefficient of friction (CoF) are shown in Fig. 8. The non-parallel interaction tendencies suggest that linked filler–surface mechanisms, as opposed to separate elements, control friction behavior.

Abrasive grit size and MCC content interact most strongly. Due to severe asperity interlocking and fiber exposure, unfilled composites show high and unstable CoF at coarse grits, while MCC-filled systems show dramatically reduced and stabilized friction, which is explained by lower interfacial shear resistance and transfer-film development. CoF values converge across filler levels as grit size increases, indicating a trend toward surface polishing and decreased mechanical interlocking. While unfilled composites continue to be more friction-sensitive, the filler–load interaction demonstrates that CoF reduces with increasing load for MCC-filled composites, indicating improved debris compaction and tribo-layer stabilization. Like this, the filler–distance interaction verifies that early production of a coherent tribo-film in MCC-filled systems causes a quick shift from running-in to steady-state friction. All things considered, Fig. 8 shows that MCC filler is a crucial factor for friction stability under changing operating circumstances.

#### Interaction effects on surface roughness

**Fig. 9:**
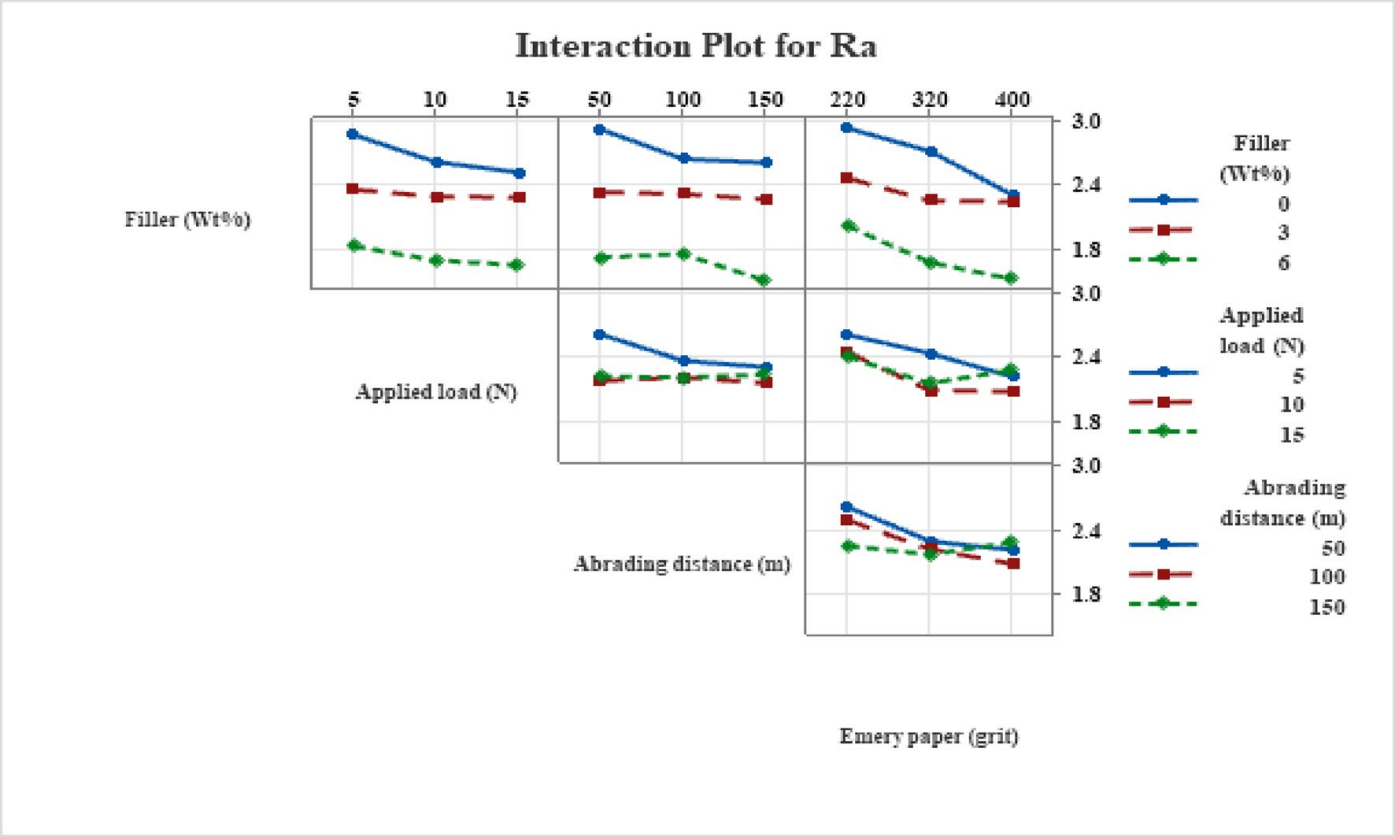
Interaction plot for surface roughness (Ra).

The effects of MCC filler content, abrasive grit, applied force, and abrading distance on surface roughness (Ra) are shown in Fig. 9. The interaction trends show that filler reinforcement and abrasive severity work together to control surface evolution. There is a noticeable filler-grit relationship, with unfilled composites showing high Ra at coarse grits because of deep ploughing and fiber exposure, while MCC-filled systems show much less roughness because of better resistance to plastic deformation and controlled fiber protrusion. For all composites, Ra decreases with increasing grit number; MCC-filled systems show improved surface refinement.

The filler–load interaction shows that although greater loads cause a little increase in roughness because of localized matrix and fiber degradation, moderate loads encourage surface smoothing in MCC-filled composites through debris compaction. By diffusing stresses, the hybrid hemp-bamboo woven architecture reduces extreme roughening. Similarly, in MCC-filled composites, the filler-distance interaction exhibits progressive smoothing and tribo-layer stability, but unfilled systems retain greater roughness. Overall, Fig. 9 demonstrates how MCC filler and hybrid weave reinforcement work together to reduce surface roughness in abrasive environments.

The interaction plots show that hybrid hemp-bamboo fabric stabilizes mechanical response across parameters, tribo-layer formation is a crucial governing mechanism made possible by optimized processing (vacuum bagging and compression molding), and MCC filler effectiveness is most noticeable under extreme abrasive and loading conditions across wear loss, CoF, and Ra.

### Contour plots

**Fig. 10:**
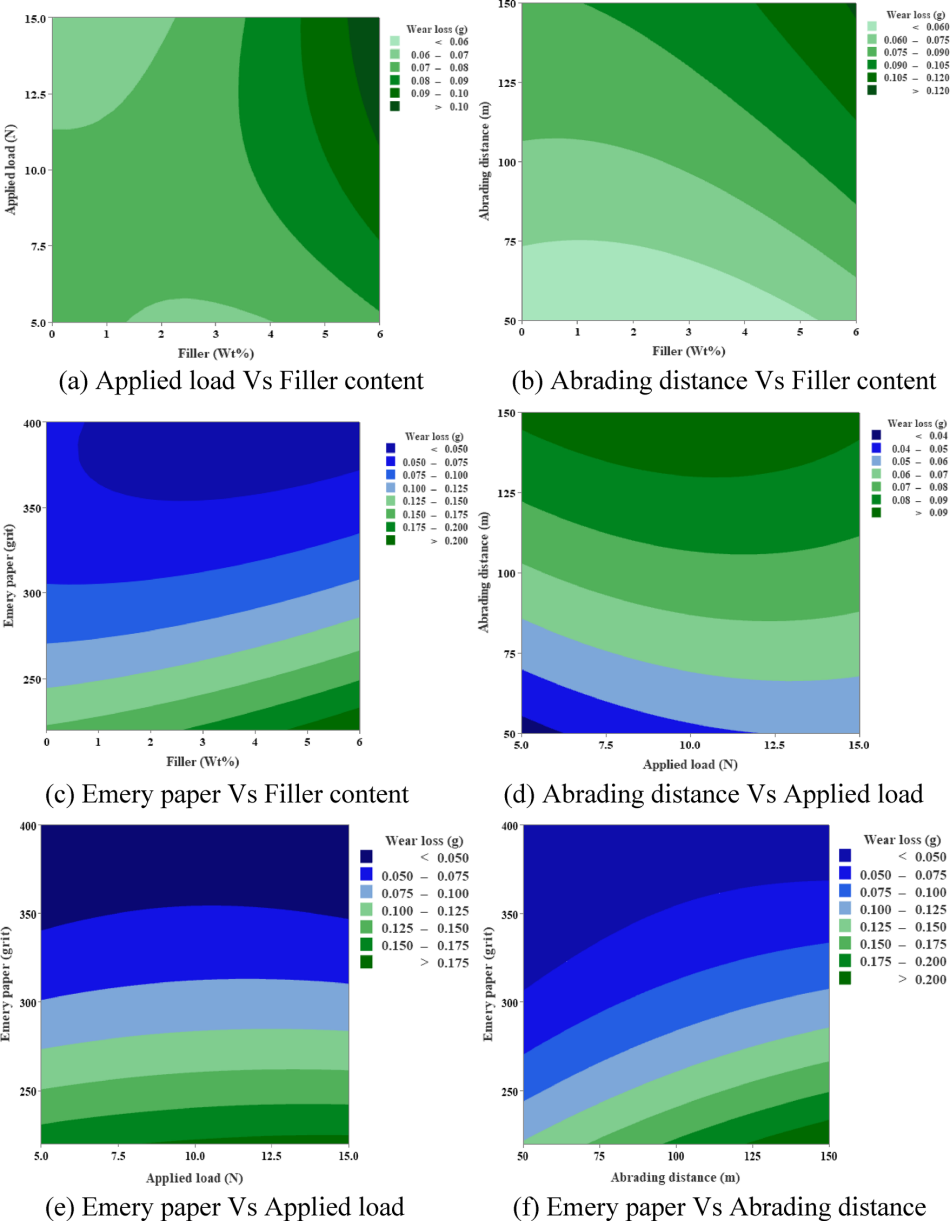
Contour plots for wear loss.

Figure 10 shows contour plots for wear loss. These plots show that wear increases steadily with applied load (250–400 N), abrading distance (50–150 m), and emery paper grit, reaching up to 0.20 g under severe conditions. Increasing the amount of MCC filler (0–6 wt%) consistently lowers wear loss, with the lowest amounts seen at 5–6 wt% across all parameters (0.04–0.06 g). This trend shows how the filler can make Hemp + Bamboo epoxy composites more resistant to wear by spreading the load more evenly and reducing the amount of material that needs to be removed.

**Fig. 11:**
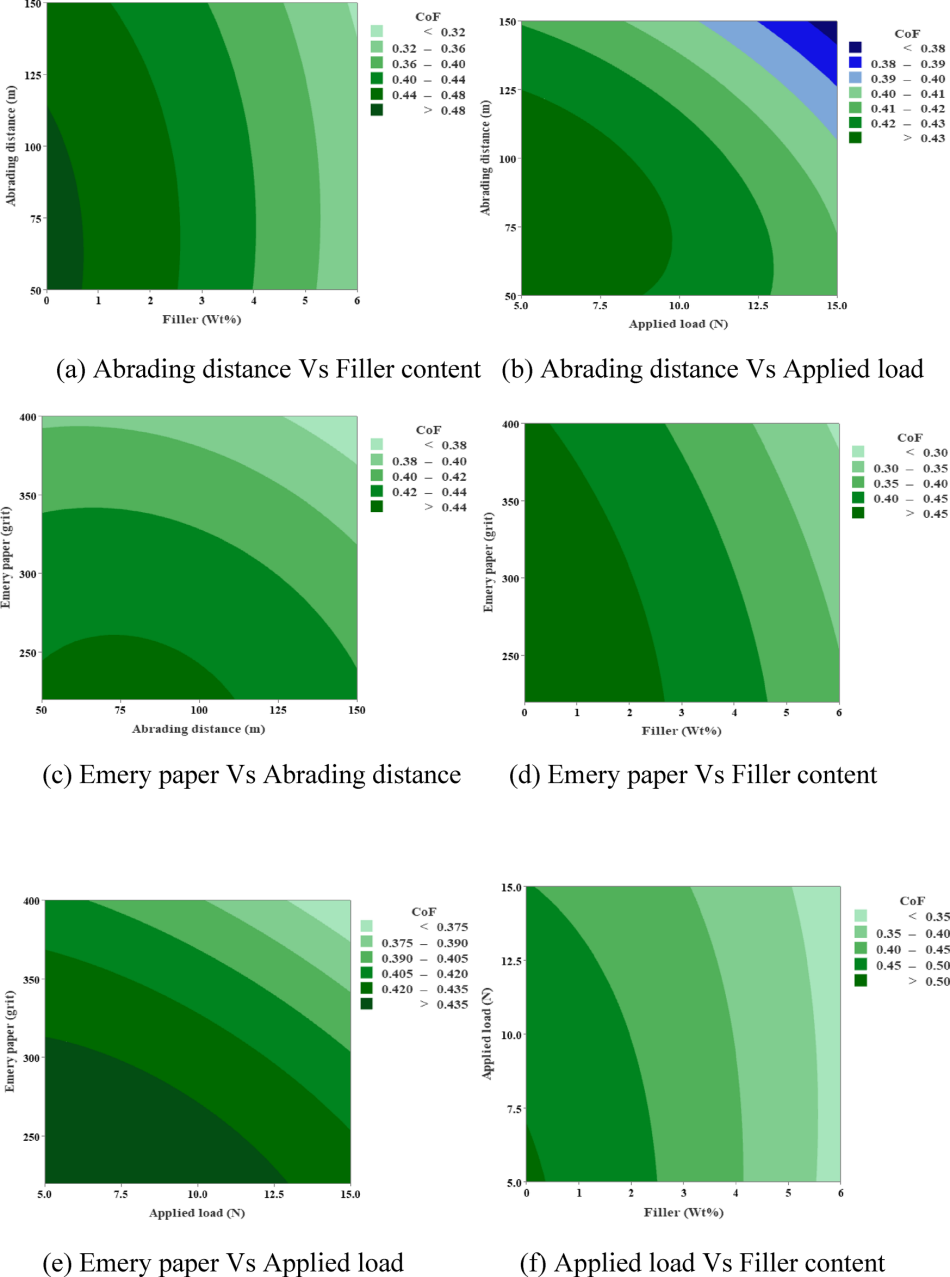
Contour plots for coefficient of friction.

Figure 11 shows coefficient of friction (CoF) contour plots that show CoF values going from 0.30 to 0.35 at low applied loads and distances to a maximum of 0.48 at high abrading distances (up to 150 m) and loads. The amount of filler has a moderate effect on CoF, with values stabilizing around 0.40 at intermediate levels (2–4 wt%) and showing slight increases at extremes. Interactions between Emery paper grit and load are the most important, which means that coarser grits make these hybrid composites more responsive to friction.

**Fig. 12:**
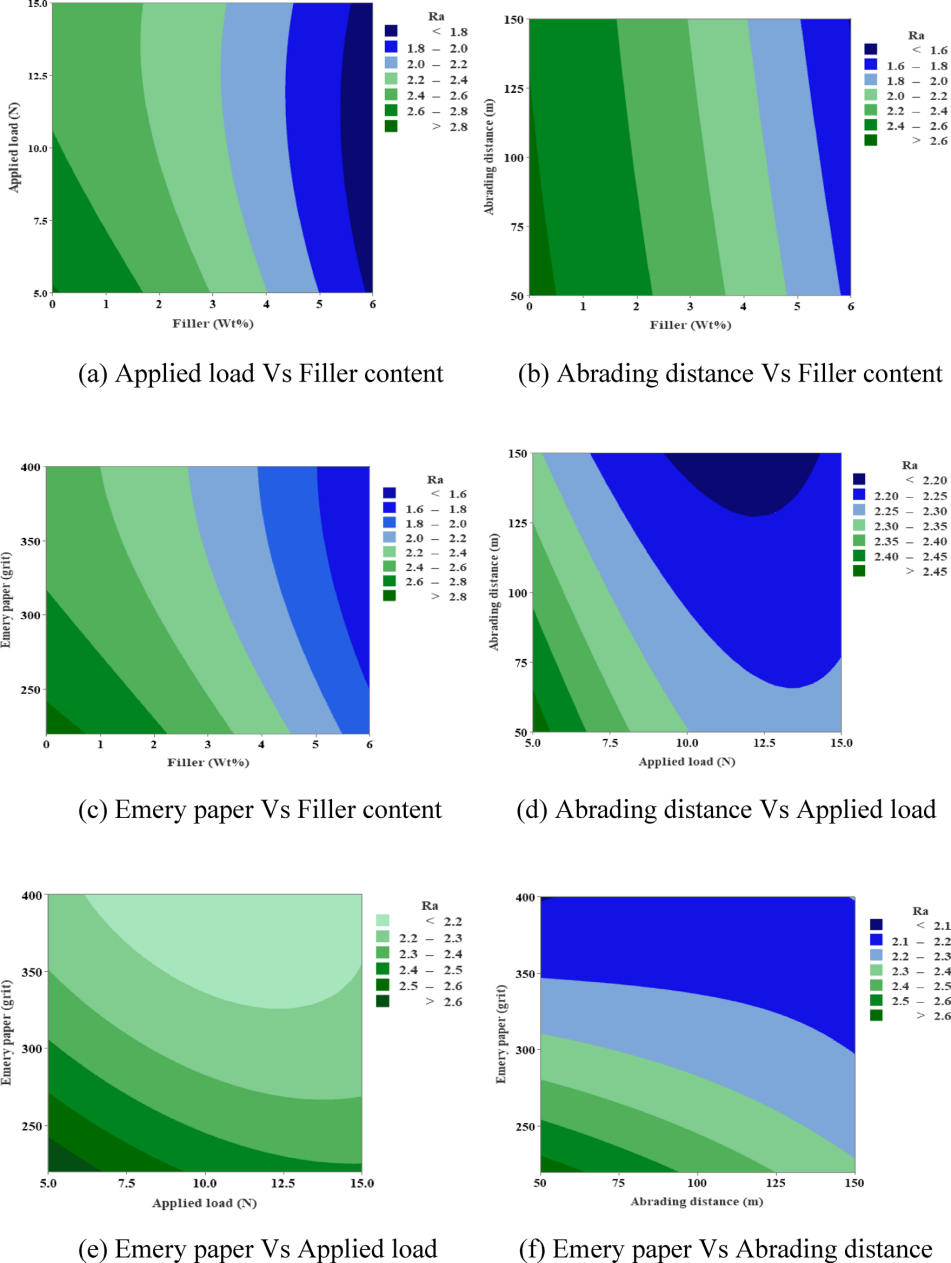
Contour plots for surface roughness.

Figure 12 shows contour plots of surface roughness (Ra). They show that Ra goes up from 1.6 to 2.8 as the applied load, abrading distance, and Emery grit goes up. The highest values are at 400 N and 150 m. Adding MCC filler lowers Ra effectively, especially when the weight% is higher (4–6%). Under the best conditions, it keeps value below 2.2. This mitigation shows that the composites have better surface integrity because the filler makes them stronger against damage from abrasives.

### Regression analysis of wear loss

To measure the correlation between the experimental parameters and the resulting wear loss, a multiple linear regression model was developed. The following is the expression for the prediction equation:

1 $$\begin{aligned} Wear{\text{ }}loss{\text{ }}\left( g \right){\text{ }} & = {\text{ }}0.2772{\text{ }} + ~0.00364~Filler~\left( {Wt\% } \right){\text{ }} + ~0.00066~Applied~load~\left( N \right) + \\ ~ & 0.000452~Abrading~dis\tan ce~\left( m \right){\text{ }} - ~0.000802~Emery~paper~\left( {grit} \right) \\ \end{aligned}$$

In line with Eq. (1), wear loss increases as filler content, applied load, and abrading distance increase because of increased surface pressure and cumulative material removal. Higher grit numbers, on the other hand, cause it to decrease since surface damage is less severe when abrasive particles are finer.

Tests of two-body abrasive wear across specific factor-level combinations were used to corroborate the statistical findings. The accuracy of the regression model was carefully evaluated by figuring out the ideal values for every test parameter.

**Table 6:** Confirmation test for wear loss.

Trails	Filler (wt%)	Load (*N*)	Abrading distance (m)	Emery paper (grit)
1	0	12	120	320
2	3	12	120	320
3	6	12	120	320

Wear loss conformance test requirements are listed in Table 6 and verification tests are performed to validate the experimental wear loss results. The wear loss was calculated using regression Eq. (1) and a pin-on-disc tribometer under conformance test circumstances. contrasting and calculating experimental wear loss results with regression model wear loss. As a result, the multiple regression equation that was derived relates the composite wear evaluation with a respectable level of precision.

**Table 7:** Confirmation test result of polypropylene different 20wt% of carbon fibers.

Trails	Experimental wear loss (g)	Regression model Wear loss (g)	% Error
1	0.0859	0.0827	3.73
2	0.0987	0.0936	5.17
3	0.1098	0.1046	4.74

#### Discussion of confirmation test findings and model validation

The predicted accuracy of the regression model is confirmed by the conformance tests for MCC filler-integrated hybrid H/B F-Ep composites, which are summarized in Table 7. With low uncertainties of 3.73–5.17%, the experimental wear loss values (0.0859–0.1098 g) closely match the model predictions (0.0827–0.1046 g).

The model’s dependability for wear forecasting under various scenarios is confirmed by the maximum errors (5.19–4.78%) falling within allowable bounds (< 6%). With small residuals suggesting no systematic bias, this tight alignment highlights the robustness of the ANOVA-derived equations (high R^2^ from Tables 3, 4 and 5). In line with earlier hybrid composite research, this validation facilitates real-world implementation for filler content optimization in abrasive conditions.

### Worn surface morphology

**Fig. 13:**
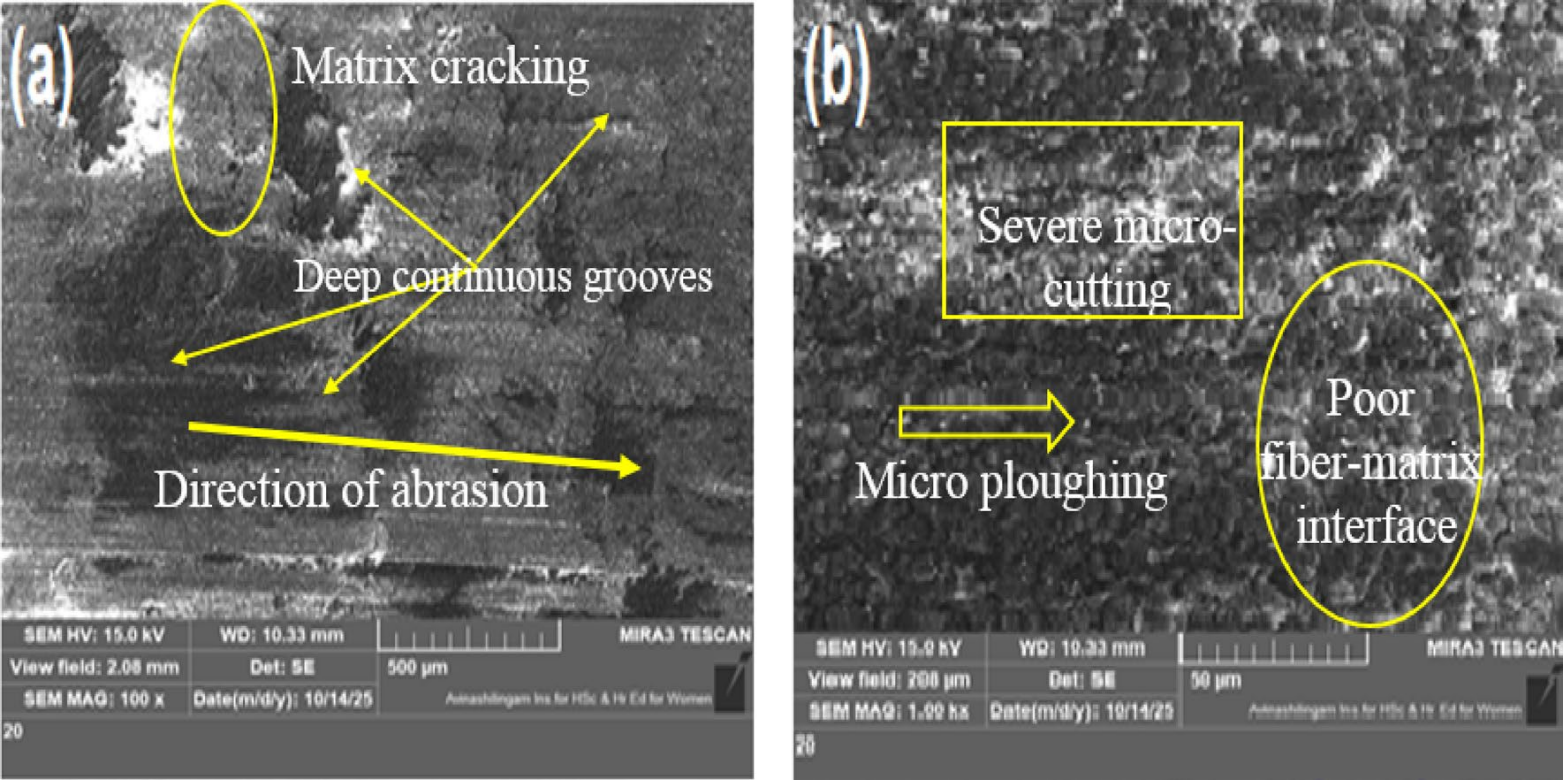
Worn surface micrographs of unfilled H/B F-Ep sample (10 N, 100 m, 400 grit).

**Fig. 14:**
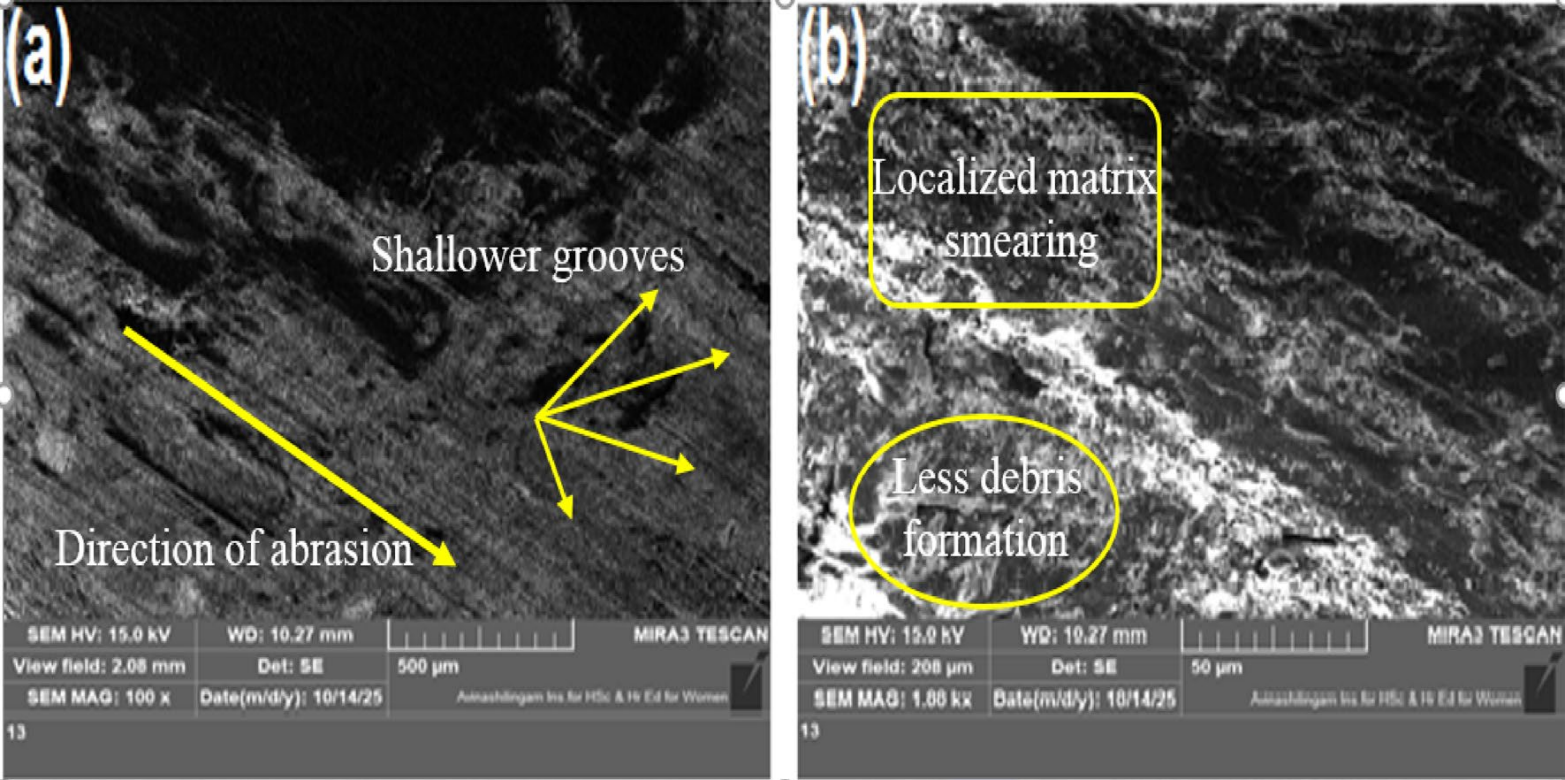
Worn surface micrographs of 3 wt% MCC filled H/B F-Ep sample (10 N, 100 m, 400 grit).

**Fig. 15:**
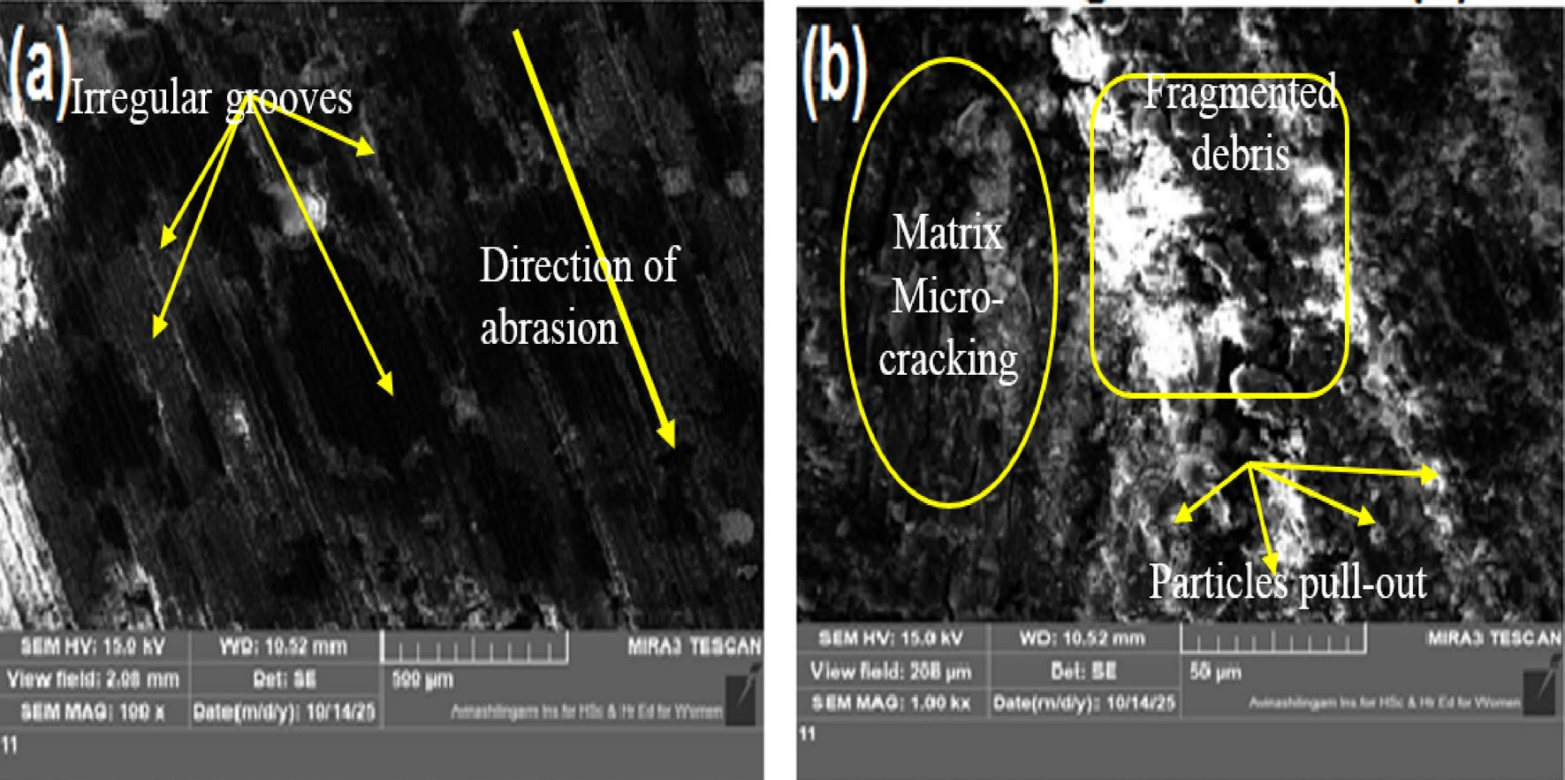
Worn surface micrographs of 6 wt% MCC filled H/B F-Ep sample (10 N, 100 m, 400 grit).

SEM micrographs of the worn surfaces (Figs. 13, 14 and 15) show that as the MCC concentration increases, abrasive wear mechanisms clearly change. The unfilled hemp-bamboo/epoxy composite (H/b F-Ep; Fig. 13a–b) has the highest wear loss (0.0403 g), CoF (0.44), and surface roughness (Ra = 2.31 μm) due to its deep, continuous grooves, severe micro-cutting, matrix tearing, and fiber pull-out, which indicate dominant micro-ploughing and unstable fiber–matrix interfacial failure.

Shallower grooves, less debris formation, and localized matrix smearing are the results of incorporating 3 wt% MCC (Fig. 14a–b), indicating a shift toward mild abrasive wear with improved load sharing and the formation of a mechanically stable tribolayer; this morphology correlates with the minimum wear loss (0.0321 g) and reduced CoF (0.40). The worn surface exhibits irregular grooves, matrix micro-cracking, and fragmented debris under increased MCC loading (6 wt%, Fig. 15a–b), suggesting micro-fracture-assisted abrasion and particle pull-out, where agglomerated MCC functions as secondary abrasives. Surface smoothing causes the CoF (0.27) and surface roughness (Ra = 1.52 μm) to drop, but wear loss (0.0376 g) rises, indicating the beginning of debris-assisted abrasion. Overall, the SEM results show that while a larger filler content encourages brittle damage processes, 3 wt% MCC offers the best resistance to two-body abrasive wear by inhibiting severe micro-cutting.

### Optimization with multiple responses

**Fig. 16:**
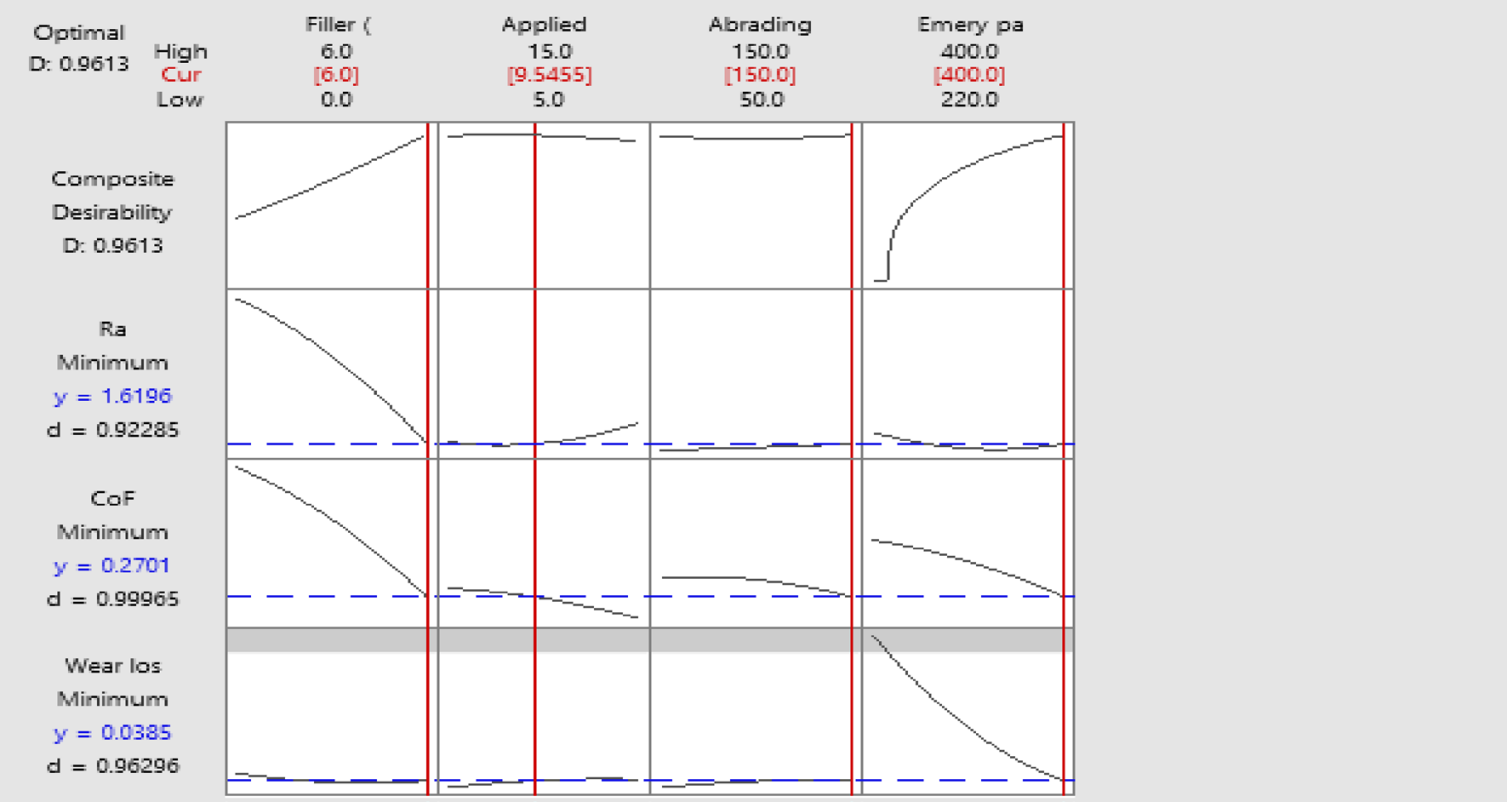
Multi-response optimization plot for minimizing cumulative surface roughness, linear wear, and coefficient of friction.

The two-body abrasive wear behavior of H/B F-Ep composites containing 0, 3, and 6 wt% MCC was analyzed and optimized using RSM depicted in Fig. 16, which is based on a four-factor, three-level Box–Behnken Design (BBD). An optimal condition was found by multi-response desirability analysis at about 3 wt% MCC, 400-grit emery paper, 150 m of abrading distance, and about 10 N applied load. This resulted in minimal wear loss (0.0385 g), low CoF (0.27), and decreased surface roughness (1.62 μm), with an overall desirability of 0.96. Intermediate MCC loading successfully stabilizes the fiber–matrix interface and reduces severe abrasive damage, as confirmed by the adjusted parameters’ good agreement with SEM-based wear mechanism analysis. Therefore, a strong framework for maximizing the tribological performance of natural fiber hybrid epoxy composites under two-body abrasive wear circumstances is provided by the combined RSM–SEM technique.

## Conclusion

This study identifies micro-crystalline cellulose (MCC) filler as a critical regulator of tribological responses in hybrid hemp/bamboo-epoxy composites by showcasing its dual function of preventing wear development and highlighting optimal loading thresholds to avoid agglomeration-induced degradation. Grit size and sliding distance are identified as the primary wear drivers, in contrast to filler dominance in friction and roughness management. Through significant quadratic and interaction effects, it also draws attention to the nonlinear, severity-dependent wear dynamics.

A robust, statistically validated framework for multi-objective tribological optimization in sustainable composites is provided by the combination of RSM and ANOVA. This framework is further strengthened by SEM confirmation of wear mechanisms, which range from severe micro-ploughing in neat matrices to stable tribo-layers at tuned MCC levels. Using multi-response desirability analysis, useful process envelopes are produced for practical implementation.

Extrapolation to three-body or erosive regimes is limited by the predominant focus on two-body abrasion; lack-of-fit in surface roughness modeling indicates possible higher-order nonlinearities that quadratic RSM is unable to handle. Fiber treatment variations and long-term cyclic fatigue under dynamic loads are not included in the scope.

Examine hybrid fillers (such as hard particles with MCC) and fibers treated with alkali or silane in a variety of multi-body wear modes. For more accurate predictions, use machine learning or cubic/polynomial models. In conjunction with scaled structural prototypes, validate under service-simulating conditions such as fluctuating temperature and humidity and evaluate life-cycle biodegradability.

## Data Availability

The authors declare that the data supporting the findings of this study are available within the paper.
